# Sodium stibogluconate loaded nano-deformable liposomes for topical treatment of leishmaniasis: macrophage as a target cell

**DOI:** 10.1080/10717544.2018.1494222

**Published:** 2018-08-14

**Authors:** M. Junaid Dar, Fakhar Ud Din, Gul Majid Khan

**Affiliations:** Department of Pharmacy, Faculty of Biological Sciences, Quaid-i-Azam University, Islamabad, Pakistan

**Keywords:** Cutaneous leishmaniasis, sodium stibogluconate, transfersomes, nano-deformable liposomes, macrophage uptake

## Abstract

Topical drug delivery against cutaneous leishmaniasis (CL) signifies an effective alternate for improving the availability and reducing the toxicity associated with the parenteral administration of conventional sodium stibogluconate (SSG) injection. The basic aim of the study was to develop nano-deformable liposomes (NDLs) for the dermal delivery of SSG against CL. NDLs were formulated by a modified thin film hydration method and optimized *via* Box–Behnken statistical design. The physicochemical properties of SSG-NDLs were established in terms of vesicle size (195.1 nm), polydispersity index (0.158), zeta potential (−32.8 mV), and entrapment efficiency (35.26%). Moreover, deformability index, *in vitro* release, and macrophage uptake studies were also accomplished. SSG-NDLs were entrapped within Carbopol gel network for the ease of skin application. The *ex vivo* skin permeation study revealed that SSG-NDLs gel provided 10-fold higher skin retention towards the deeper skin layers, attained without use of classical permeation enhancers. Moreover, *in vivo* skin irritation and histopathological studies verified safety of the topically applied formulation. Interestingly, the cytotoxic potential of SSG-NDLs (1.3 mg/ml) was higher than plain SSG (1.65 mg/ml). The anti-leishmanial activity on intramacrophage amastigote model of *Leishmania tropica* showed that IC_50_ value of the SSG-NDLs was ∼ fourfold lower than the plain drug solution with marked increase in the selectivity index. The *in vivo* results displayed higher anti-leishmanial activity by efficiently healing lesion and successfully reducing parasite burden. Concisely, the outcomes indicated that the targeted delivery of SSG could be accomplished by using topically applied NDLs for the effective treatment of CL.

## Introduction

Cutaneous leishmaniasis (CL) is a neglected parasitic disease caused by the obligate intracellular protozoa belonging to the genus *Leishmania* and transmitted *via* the bite of female sandflies. More than 15 species of *Leishmania* are responsible for CL and reside within the macrophages present in the skin dermis. These parasites instigate a range of distinct clinical manifestations varying from small nodules to large plaques and ulcers (Reithinger et al., 2007). Every year, almost 1.5 million new cases of CL are reported (Kumar et al., 2007) and is widespread in 88 countries around the globe. In sandfly, *Leishmania* parasite resides as a motile promastigote and transforms itself into a non-motile amastigote once engulfed by the host macrophages. The amastigote form has evolved to persist and multiply within the harsh environment of macrophages (Frézard & Demicheli, 2010).

Sodium stibogluconate (SSG), a pentavalent antimony (Sb) compound, is used as a standard recommended treatment for the CL. It is a high molecular weight (910.9 g/mol) SbV-gluconate complex compound having log *P* value (−3.41) and high aqueous solubility (100 mg/ml). Parenteral injection of SSG is usually accompanied by serious side effects and needs multiple injections for weeks (Kashani et al., 2007). Other drugs prescribed as the second choice of treatment are amphotericin B, paromomycin, and pentamidine, however, these are toxic, expensive, and require parenteral administration (Barrett et al., 1999).

The targeted drug delivery produces more desirable effects than parenteral administration with additional advantage of minimum side effects (Dar et al., 2017). The World Health Organization (WHO) encourages the topical treatment against CL and recommends the parenteral administration only if the topical therapy fails (WHO, 1990). Intralesional injection of antimonial drugs is considered more effective and practical which supports the development of topical formulation (Khamesipour et al., 2010). The advanced drug carriers find new opportunities in the topical delivery of anti-leishmanial drugs, which were already tested in conventional creams with poor success rate (Espuelas, 2015). In the present era, anti-leishmanial drug loaded liposomes, lipid nanocarriers, polymeric particles, and dendrimers are being given full consideration for their application in the topical treatment of CL. The effectiveness of topical treatment against CL depends on two factors: (i) the amount of drug that reaches dermis, where infected macrophages reside and (ii) the intrinsic anti-leishmanial activity of the drug (Garnier & Croft, 2002). To permeate the stratum corneum (SC), an active entity should have a low molecular weight (<500 g/mol) and a partition coefficient value between one and three (Hadgraft & Pugh, 1998). However, the physicochemical properties of SSG impair its skin permeation and therefore needs an efficient vehicle system for the skin permeation.

Intensive research over the past many years has led to the development of ultradeformable liposomes/transfersomes (Cevc, 1996). In contrast to conventional liposomes which are unable to penetrate deep into the skin and remain confined to the SC (Touitou et al., 2000), these elastic vesicles could penetrate into deeper skin layers. The transfersomes or nano-deformable liposomes (NDLs) are composed of phospholipids and an edge activator (single chain surfactant) which provides a high radius of curvature. This modification destabilizes the rigid lipid bilayer which in turn enhances the deformability of the vesicles (Cevc, 1996). NDLs could be loaded with a wide range of low molecular weight drugs like 5-fluorouracil (El Maghraby et al., 2001) as well as with high molecular weight drugs such as insulin (Cevc et al., 2002). Diagrammatic illustration of mechanism governing the SSG-NDLs permeation in the skin *via* deformation/reformation mechanism and uptake by dermal macrophages is illustrated in Supplemental Figure S1.

The present work focuses on the design and development of SSG-loaded NDLs (SSG-NDLs) gel formulation for the dermal delivery having improved activity against CL with minimum side effects. To the best of our knowledge, this study is the first depiction of the NDLs as a carrier of SSG in the treatment of CL.

## Materials and methods

### Materials

SSG was generously gifted by Star Laboratories, Pakistan. Phospholipon^®^ 90G (soy phosphatidylcholine) was a kind gift from Lipoid AG, Switzerland. Tween-80, RPMI-1640, fluorescein isothiocyanate (FITC), and Giemsa stain were acquired from Sigma Aldrich, Germany. Cellu Sep^®^ dialyzing membrane (12–14 kDa) was purchased from Membrane Filtration Products (Texas, USA). All other reagents were of pure analytical grade.

### Preparation and purification of SSG-NDLs

SSG-NDLs were prepared by using thin film hydration method (El Zaafarany et al., 2010) with some modifications. Briefly, Phospholipon^®^ 90G and Tween-80 were dissolved in chloroform:methanol (1:1) mixture and evaporated by using rotary evaporator under vacuum at 40 ± 1 °C to develop a clear film. The film was hydrated with SSG solution in phosphate buffered saline (PBS) of pH 7.4 for 1 h at 60 ± 1 °C. The prepared formulation was then manually extruded five times through polycarbonate membrane filters (450 and 200 nm). The unentrapped drug was removed by dialysis at 4 °C and stored in air tight glass container at 4 °C for further analysis.

### Experimental design for the optimization of SSG-NDLs

A three-level, three-factor Box–Behnken design (BBD) was exercised to optimize the formulation variables, using response surface methodology (RSM). Three different independent variables were assessed: SSG concentration (*X*_1_), amount of phospholipid (*X*_2_), and percentage of edge activator (*X*_3_). The vesicle size (*Y*_1_) and entrapment efficiency (*Y*_2_) were chosen as the dependent variables. The details of independent and dependent variables are shown in Supplemental Table S1. The polynomial equations correlating the independent and dependent variables were generated in order to achieve optimum vesicle size (VS) and high entrapment efficiency (EE) using Design Expert (Trial Version 10, State-Ease Inc., MN). A total of 15 SSG-NDLs formulations were prepared, as indicated in Table 1. The optimum size range for particle uptake by macrophages is >100 nm, therefore constraint regarding VS of >100 nm was applied along with maximum EE%.

**Table 1.** Composition, entrapment efficiency, and vesicle size of SSG-NDLs.

Run	Independent variables			Dependent variables	
	*X*_1_ (mg/ml)	*X*_2_ (mg)	*X*_3_ (% w/w)	*Y*_1_ (nm ± SD)	*Y*_2_ (% ± SD)
F1	50	300	5	100 ± 5.23	2.7 ± 0.71
F2	50	200	10	89.63 ± 6.12	4.3 ± 1.92
F3	75	300	10	137.5 ± 3.8	17.23 ± 4.83
F4	50	400	10	157.7 ± 7.36	13.9 ± 5.36
F5	75	400	15	231.8 ± 9.72	20.3 ± 3.95
F6	50	300	15	69.13 ± 4.53	9.18 ± 5.63
F7	100	400	10	195.1 ± 3.67	35.26 ± 6.28
F8	75	400	5	213.1 ± 8.41	24.8 ± 4.19
F9	75	300	10	153.7 ± 3.84	21.6 ± 7.47
F10	75	200	15	35.37 ± 2.73	4.5 ± 2.91
F11	75	200	5	187.9 ± 6.18	18.2 ± 5.69
F12	100	300	5	114.4 ± 4.36	28.3 ± 7.36
F13	100	200	10	92.38 ± 5.52	19.4 ± 3.94
F14	75	300	10	170.1 ± 7.29	14.5 ± 5.89
F15	100	300	15	58.7 ± 3.81	25.7 ± 6.77

*X*_1_: SSG concentration; *X*_2_: amount of phospholipid; *X*_3_: percentage of edge activator; *Y*_1_: vesicle size; *Y*_2_: entrapment efficiency.

### Physicochemical characterization of NDLs

The optimized SSG-NDLs were characterized in terms of mean VS, polydispersity index (PDI), zeta potential (ZP), and EE%. The average VS, PDI, and ZP of NDLs (before and after dialysis) were established by using Zetasizer ZS90 (Malvern instruments, UK). Prior to measurement, 10 µl of NDLs were diluted up to 5 ml with double distilled water. The direct method was used for the determination of EE% by separating the unentrapped SSG from NDLs *via* exhaustive dialysis at 4 °C (Frézard et al., 2000). Briefly, SSG-NDLs was transferred into the dialysis bag (molecular weight cut off 12–24 kDa), dropped in PBS (pH 7.4), and stirred magnetically. The dialysis medium was changed after every 2 h and analyzed for SSG contents using atomic absorption spectrophotometer (AAS), till no SSG was detectable. After dialysis, the formulation was lysed by dissolving it in pure nitric acid, followed by heating the sample till it was dried completely. Subsequently, HCl:water solution in 1:1 ratio was added, boiled for 1 h, and EE% was figured by using following equation:

$$\text{EE}\left(\%\right)=\left(\frac{A_{2}}{A_{1}}\right)*100$$

where *A*_2_ is the amount of SSG entrapped and *A*_1_ is the total amount of SSG added.

Surface morphology of SSG-NDLs was examined by transmission electron microscope (TEM) (Philips CM12, Netherlands). Diluted sample was placed on copper grids, followed by negative staining with 1% phosphotungstic acid and analyzed.

### Deformability of SSG-NDLs

The deformability of nano-vesicles was determined by a method reported elsewhere (Pathak et al., 2016) with some modifications. Briefly, NDLs were extruded through a disposable 100 nm membrane filter. Followed by extrusion, the weight of suspension and mean VS were measured. The deformability index of NDLs was then computed by using following equation:

$$D=J*{\left(\frac{r_{v}}{r_{p}}\right)}^{2}$$

where *D* is the deformability, *J* is the weight of suspension, *r*_v_ is the VS after extrusion, and *r*_p_ is the pore size of filter.

### Preparation of SSG-NDLs gel

The optimized SSG-NDLs were not viscous enough to stay on the skin. Therefore, the vesicles were added into 1% (w/v) Carbopol 934 gel to make it rheologically acceptable. Carbopol was slowly dispersed in the distilled water with continuous stirring for 3–4 h. The SSG-NDLs were added and mixed completely. Finally, triethanolamine was added dropwise to neutralize the mixture (Kumar Jain & Puri, 2014).

### Physicochemical and rheological evaluation of SSG-NDLs gel

The blank and SSG-NDLs gel were evaluated in terms of clarity, appearance, pH, drug content, and rheological properties. The pH of gel formulation was determined by a pH meter after dissolving 1 g of gel in 50 ml of distilled water. The drug content of gel formulations was measured by using AAS after adding 1 g gel in HCl:water (1:1) solution, followed by sonication at 80 °C for 30 min. The rheological parameters were measured by a viscometer at room temperature. The measurements were accomplished at a constant shear speed (40 rpm) and varying shear speeds (2–120 rpm) with a 64-number spindle. The apparent viscosity of the gel formulations at each speed was recorded and the flow pattern was established from the rheogram (Singh et al., 2017).

### *In vitro* drug release and release kinetic study

The drug release from SSG solution, SSG-NDLs, and SSG-NDLs gel were determined in sodium acetate buffer (pH 5.5) and PBS (pH 7.4) as the release media to imitate normal physiological and endosomal pH of the macrophages, respectively (Nahar & Jain, 2009). Briefly, formulations were placed inside a dialysis bag and dialyzed against release media maintained at 37 ± 1 °C in a shaker water bath. The samples were drawn at pre-determined time and replaced with buffer to maintain the sink condition. The cumulative percentage drug release was calculated and graph was created. Moreover, drug release data were subjected to different kinetic models. The regression analysis was then executed and best fit correlation was achieved.

### *Ex vivo* permeation and drug deposition studies

The freshly excised rat skin was placed on a locally fabricated Franz diffusion cell having effective permeation area of 0.77 cm^2^ with 5.2 ml PBS (pH 7.4) capacity of receiving compartment and was set at constant stirring rate of 300 rpm. The temperature of system was maintained at 32 ± 1 °C throughout the experiment to simulate the skin condition, as reported previously (Mir-Palomo et al., 2016). The donor compartment was filled with SSG solution, SSG-NDLs, and SSG-NDLs gel equivalent to 8 mg of SSG under non-occlusive and open hydration protocol (El Maghraby et al., 2001). The samples were drawn at predetermined time and replaced with fresh medium. The cumulative amount of SSG permeated per unit area was plotted against time. The flux (*J*_max_) at 24 h and the enhancement ratio (ER) were calculated by using the equations:

$$J_{\max}=\frac{\text{Amount}\text{of}\text{permeated}\;\text{drug}}{\text{Time}*\text{Area}\text{of}\text{the}\;\text{membrane}}$$

$$\text{ER}=\frac{J_{\text{max}\;\text{of}\;\text{NDLs}}}{J_{\text{max}\;\text{of}\;\text{control}}}$$

The concentration of SSG detained in the SC and epidermis/dermis layers was also evaluated. At the end of *ex vivo* permeation study, the skin samples were recovered, washed, and blot dried. The SC was separated using tape stripping method as mentioned elsewhere (Montanari et al., 2010). The skin pieces were stripped with 20 pieces of adhesive tape, covering the entire surface of the skin samples. All the tapes were collected, placed in a beaker and SSG was extracted by boiling the tapes in a mixture of HCl:water (1:1), followed by SSG content determination. The remaining skin section was chopped into pieces, meshed, homogenized, and SSG amount was determined.

### *Ex vivo* penetration study with fluorescent dye

Fluorescein isothiocyanate (FITC)/SSG-NDLs formulation was used for analyzing *ex vivo* penetration study. FITC/SSG-NDLs were prepared and applied equally over the rat skin placed on the Franz diffusion cell (conditions of *ex vivo* permeation study was maintained) while untreated skin section was used as a control to establish the normal tissue fluorescence. After 24 h, the skin surface was carefully washed and sectioned using cryostat microtome. The longitudinal section (5 μm) was mounted on the slides and visualized without any additional treatment or staining using a fluorescent microscope.

### Evaluation of skin structure after SSG-NDLs treatment

The skin characteristics were investigated by attenuated total reflectance-Fourier transform infrared spectroscopy (ATR-FTIR) and differential scanning calorimetry (DSC) after formulation treatment. The permeation enhancers may induce structural changes in the epidermis region, specially SC. The epidermis of rat skin was separated by placing it in water (60 °C) for 2–3 min and then peeled off. The separated epidermis was placed on Franz diffusion cell and treated with the SSG-NDLs for 5 h. The epidermis was then washed and subjected to ATR-FTIR and DSC after blot drying. The untreated epidermis was used as a control. The molecular vibrations of skin lipids were studied by ATR-FTIR over the range of wave number 4000–400 cm^−1^ while thermal analysis of isolated epidermis was evaluated by DSC.

### *In vivo* skin irritation and histopathological study

The study was accomplished according to a reported procedure (Draize et al., 1944), with slight modifications. The rats were used in this study and the handling protocol was approved by the Quaid-i-Azam university ethics committee (BEC-FBS-QAU2018-11). The animals were divided into three groups (*n* = 5) and examined for any skin reaction after topical application of SSG-NDLs gel and 0.8% formalin solution while one group was provided with no treatment. The primary irritancy index (PII) was estimated by adding the edema and erythema scores of each group. The scores were classified as PII <2 (nonirritant), 2–5 (irritant), and 5–8 (highly irritant). The irritation profile was further verified by histopathological analysis of skin. The skin samples of each group were sectioned using cryostat microtome and examined under a microscope.

### Stability study

The physical stability of the optimized SSG-NDLs and SSG-NDLs gel was estimated at 4 and 25 °C for 60 days in terms of monitoring changes in VS, PDI, ZP, EE%, precipitation, and phase separation. For this, samples were collected at pre-determined days and analyzed for important physicochemical parameters.

### Macrophage cytotoxicity assay

To estimate the cytotoxicity of plain SSG solution and SSG-NDLs, an *in vitro* assay was performed in the peritoneal macrophages (PMs) of mice, using trypan blue exclusion assay. Briefly, 1.5 ml of 3% (w/v) sterile thioglycolate was inoculated into the peritoneal cavity of BALB/c mice. After 5 days, the mice were euthanized and 5 ml of ice cold RPMI-1640 was injected in the peritoneal cavity. The peritoneal exudate was then recovered followed by centrifugation at 3000 rpm for 10 min. The pellet obtained was then suspended in RPMI-1640, supplemented with 1% of a 100U/ml penicillin, 10% FBS, and 100 µg/ml of streptomycin solution. PMs were adjusted to 2 × 10^4^ cells/well (200 µl/well) and incubated in 24-well plates having 13 mm coverslips for 24 h under 5% CO_2_. The plain SSG solution and SSG-NDLs at different concentrations were added to the wells and again incubated for 24 h. Trypan blue solution 0.5% (w/v) was prepared, having 0.06% (w/v) monobasic potassium phosphate and 0.9% sodium chloride. It was added to SSG solution and SSG-NDLs treated PMs wells followed by incubation for 15 min. Concentration of the drug that caused 50% mortality in macrophages (CC_50_) was then accomplished by using following equation:

$$\text{Cell}\;\text{viability}\left(\%\right)=\{\frac{\text{Total}\;\text{viable}\;\text{cells}\left(\text{unstained}\right)}{\text{Total}\;\text{cells}\left(\text{stained}+\text{unstained}\right)}\}*100$$

It was noted that SSG itself affect the MTT assay, therefore standard trypan blue exclusion assay was performed.

### Qualitative and quantitative macrophage uptake study

In order to study the uptake of SSG-NDLs in PMs, a separate set of experiments was conducted. The PMs isolated *via* above method was seeded into culture well plate along with FITC/SSG-NDLs in order to determine qualitative uptake of vesicles, while simple SSG-NDLs was used as a control. The cells were then placed in CO_2_ incubator at 37 °C for 30 min and the attached cells were washed, followed by observation under a fluorescent microscope.

In order to determine the cellular uptake of NDLs quantitatively, the pure SSG solution and SSG-NDLs (300 µg/ml) were seeded in culture well plate. The plate was left for 24 h and then washed with PBS. The adhered cells were obtained by scraping them off from slides and centrifuged. The pellet was re-dispersed in methanol, sonicated, and centrifuged again, followed by determination of SSG through AAS.

### Anti-leishmanial activity on intramacrophage amastigote model

Anti-leishmanial activity of plain SSG and SSG-NDLs was evaluated by using an intramacrophage amastigote model. Previously isolated PMs were seeded into 24-well culture plate with coverslips at a density of 2 × 10^4^ cells/well, followed by incubation in CO_2_ incubator and permitted to adhere. The cells were washed thrice and attached cells were then infected with metacyclic promastigotes of *Leishmania tropica* (KHW23) at 10:1 (promastigote: macrophages) ratio. Infected cells were incubated for 24 h at 37 °C and then washed twice to remove un-phagocytosed promastigotes, followed by incubation of infected macrophages with different concentration of SSG and SSG-NDLs. After 24 h, slides were fixed with methanol, stained with 10% Giemsa and visualized under a microscope. The inhibitory concentration at which 50% of amastigotes were killed (IC_50_) and selectivity index (SI) were then calculated

$$\text{Inhibition}\left(\%\right)=\left(\frac{\text{No}.\;\text{of}\;\text{amastigotes}\;\text{in}\;\text{experimental}\;\text{well}\;}{\text{No}.\;\text{of}\;\text{amastigotes}\;\text{in}\;\text{control}\;\text{well}}\right)*100$$

$$\text{Selectivity}\;\text{index}\left(\text{SI}\right)=\frac{{\text{CC}}_{50}}{{\text{IC}}_{50}}$$

### *In vivo* efficacy of formulation in BALB/c infection model of CL

Female BALB/c mice were purchased from National Institute of Health (NIH), Islamabad and housed under standard laboratory diet and conditions (25 ± 1 °C temperature and 55 ± 5% humidity). The experimental protocols were approved by the ethical committee of Quaid-i-Azam University, Islamabad (BEC-FBS-QAU2018-47). The mice were subcutaneously injected in the base of tail with parasite suspension having 2 × 10^6^ promastigotes of *Leishmania* (stationary phase) suspended in 50 µl of RPMI medium. Lesion size was measured daily and treatment was started when nodule reached 4–5 mm in size. Animals were randomly divided into three groups (*n* = 5) and treated every 24 h for 21 days with SSG-gel (20 mg/kg) and SSG-NDLs gel (20 mg/kg), while the control group was left untreated. The drug efficacy was assessed at the end of treatment by the measurement of lesion size, as the mean of tail base diameters in vertical and horizontal directions. The intensity of infection was determined by microscopic examination of stained smears at the end of experiment. Briefly, lesions were cleaned, punctured at the margins, and exudation material was smeared on a clean slide. The smear was fixed in absolute methanol, followed by staining with Giemsa. The average number of amastigotes inside 50 macrophages was calculated and infection intensity was assessed according to defined criteria (WHO, 1991), as weak (<100 amastigotes/macrophages), moderate (100–1000 amastigotes/macrophages) and severe (>1000 amastigotes/macrophages) infection.

### Statistical analysis

All the data were represented as a mean ± SD and results were statistically analyzed by One-way ANOVA, along with Dunnett’s *t*-test, which was used to determine statistical significance, using the Sigmaplot (Version 12.5), SYSTAT Software Inc, UK. Significance was specified at *p* < .05 level of probability.

## Results and discussion

### Effect of independent variables on vesicle size

The VS range of prepared SSG-NDLs is shown in Table 1, while RSM plot for the effect of independent variables on the VS is illustrated in Supplemental Figure S2. As explained previously, VS <100 nm was not advantageous for this study as vesicles may escape uptake by dermal macrophages. Moreover, it was reported that vesicles having intermediate size were efficient in penetrating the deeper layers of the skin with additional benefit of higher EE% (Verma et al., 2003). The ANOVA test for the VS suggested that the quadratic model was significant. The resulting equation in terms of coded values was

$$\text{Vesicle}\;\text{size}\;\left(Y_{1}\right)=+153.77+5.51X_{1}+49.05X_{2}-27.55X_{3}+8.66X_{1}X_{2}-6.21X_{1}X_{3}+42.81X_{2}X_{3}-50.77X_{1}^{2}+30.71X_{2}^{2}-17.43X_{3}^{2}$$

The positive value of a factor signifies a synergistic effect of that response and *vice versa*. The polynomial equation suggested that the SSG concentration (*X*_1_) had a minor and non-significant (*p* = .3773) effect. For the preparation of NDLs, the SSG was introduced in the hydration medium, which means that drug would be encapsulated within inner aqueous core (Abdelbary & AbouGhaly, 2015). The NDLs entrap same volume of hydration medium irrespective of drug concentration and this medium was kept constant. Therefore, VS was not significantly affected by the concentration of SSG in the hydration medium.

The ANOVA results described the synergistic effect of amount of phospholipid (*X*_2_) on the VS (*p* = .0003). This may be due to the presence of increased quantity of phospholipids comparative to hydration medium. In order to accommodate the inadequacy of hydration medium, the surface area may decreased and subsequently resulted in the increase of VS (Sankhyan & Pawar, 2013).

The negative coefficient of percentage of edge activator (%EA) (*X*_3_) indicated that it had a negative impact on the VS of NDLs (*p* = .0047). The increase in %EA may increase the quantity of surfactant that covers the surface of vesicles which may lower the interfacial tension and allow the formation of small vesicles (Aboud et al., 2016).

### Effect of independent variable on percentage entrapment efficiency

The ability of NDLs to encapsulate substantial amount of SSG is important for its application in the treatment of CL. The values of EE% obtained are mentioned in Table 1, while RSM plot was illustrated in Supplemental Figure S2. ANOVA test for the observed EE% data implied that the linear model was significant. The resultant equation achieved was mentioned below:

$$\text{Entrapment}\;\text{efficiency}\left(Y_{2}\right)=+17.32+9.82X_{1}+5.98X_{2}-1.79X_{3}$$

The equation showed that there was a significant positive (*p* < .0001) effect of *X*_1_ on the EE%. This effect may be due to the saturation of hydration medium with SSG that drive the drug to entrap inside the vesicles (El-Samaligy et al., 2006). Moreover, taking into account that the same volume of hydration medium was encapsulated inside the NDLs, the increased drug concentration in this medium would suggest that more quantity of drug was encapsulated.

The positive value of *X*_2_ implied that it had a positive effect on the EE% (*p* = .0008). The increased amount of vesicle forming material may enhanced the total phospholipids available for hydration, which resulted in production of higher number of NDLs, engulfing more volume of hydration (Abdelbary & AbouGhaly, 2015).

Finally, the equation revealed no significant influence of *X*_3_ on the EE% (*p* = .1985). A study found that by increasing the concentration of Tween-80 from 2% to 25%, there was insignificant (50.68–48.71%) variation in EE% (El Zaafarany et al., 2010). It might be possible that NDLs were formed at lower concentration of EA and further increase may only increase their deformability potential, with minor effect on EE%.

### Optimization and validation

The BBD was used for designing and optimization of the experimental trials. This design requires fewer experimental trials than a full factorial design. The Design Expert software predicted an optimized formula, after employing constraints on the EE% and VS. Important statistical parameters of BBD are shown in Supplemental Table S2. The suggested formulation (F7) had 100 mg/ml SSG concentration, 400 mg phospholipid, and 10% EA. The suggested formulation was prepared and assessed, while small variation in responses (predicted and observed) was observed, signifying the validity of the optimization procedure.

### Physicochemical characterization of NDLs

The VS and PDI are important parameters for permeation of drug through SC. The optimized NDLs showed mean VS around 200 nm in both dialyzed and un-dialyzed formulations while resultant distribution curve was unimodal in shape as shown in Supplemental Figure S3(a). It was believed that vesicles having ≤300 nm size can effectively deliver entrapped drug deep into deep skin layers (Verma et al., 2003). The optimized NDLs also exhibited narrow vesicle size distribution (PDI = 0.158) which improved drug permeation through SC. PDI value less than 0.2 implies a monodisperse and homogenous population. It was observed that dialyzed SSG-NDLs had a significantly higher zeta potential (Supplemental Figure S3(b)) value (−32.8 mV) as compared to un-dialyzed one (−7.66 mV). It was expected that dialyzed SSG-NDLs would display improved physical stability in comparison to un-dialyzed formulation. The magnitude of the zeta potential designates the potential stability of vesicles and as the zeta potential increase so does the stability (Honary & Zahir, 2013). TEM image of optimized SSG-NDLs demonstrated uni-lamellar structure, sealed vesicles and near to spherical shape (Supplemental Figure S3(c)). Moreover, the VS attained using TEM was in agreement with that achieved by zeta sizer.

SSG is a hydrophilic drug and vesicle-based formulation displayed a very low (6%) EE (Nieto et al., 2003) while the EE of optimized SSG-NDLs was found to be 35.26 ± 1.8%. To increase the EE of a hydrophilic drug (SSG), different strategies were used in the present study. The negatively charged vesicles were used which claimed to encapsulate antimonial compounds more efficiently than the neutral or positively charged vesicles (Frézard & Demicheli, 2010). NDLs based formulation was prepared which displayed higher EE in comparison to niosomes and liposomes (Gupta et al., 2005). The thin film hydration method was used for the preparation of NDLs in which a thin lipid film was formed on a large surface area and complete hydration of the vesicles resulted in higher EE (Jain et al., 2003). Moreover, sonication step in the preparation stage was skipped and replaced with extrusion technique. Sonication results in the breakage of vesicles and therefore decreases the EE of hydrophilic drugs (Chaudhary et al., 2013).

### Deformability of SSG-NDLs

The degree of shape transformation and stress dependent adaptability in NDLs is expressed by deformability index. The higher deformability value imparts higher skin permeation characteristic to the vesicles. The deformability indices of optimized SSG-NDLs was found to be 43. Studies indicated that Tween-80 displayed a highest deformability value due to its long, highly pliable, and non-bulky hydrocarbon chains which imparts higher flexibility to the lipid bilayer as compared to other surfactants (El Zaafarany et al., 2010).

### Physicochemical and rheological evaluation of SSG-NDLs gel

Carbopol 934 was selected as a carrier base for NDLs due to its excellent bio-adhesive properties, nonirritant nature, ability to form pharmaceutically elegant gels, and better storage stability (Kaur et al., 2015). The gel formulation exhibited neutral pH, considered ideal for topical application without having possibility of skin irritation, and had high drug content (92.13 ± 4.12%). The rheological properties of gel are important because they affect ease of skin application, adhesion, and subsequent retention at the site of application. SSG-NDLs gel and simple NDLs gel showed no significant difference (4.5 ± 0.12 and 4.9 ± 0.14 Pa s, respectively) in viscosities at the shear rate of 40 rpm. The flow curve was achieved by plotting shear speed against respective viscosity (Supplemental Figure S3(d)). The analysis of rheogram revealed a non-Newtonian pseudo-plastic flow pattern which is desirable for topical applications as it covers a maximum skin area (Kaur et al., 2015).

### *In vitro* drug release and release kinetic study

**Figure 1.**
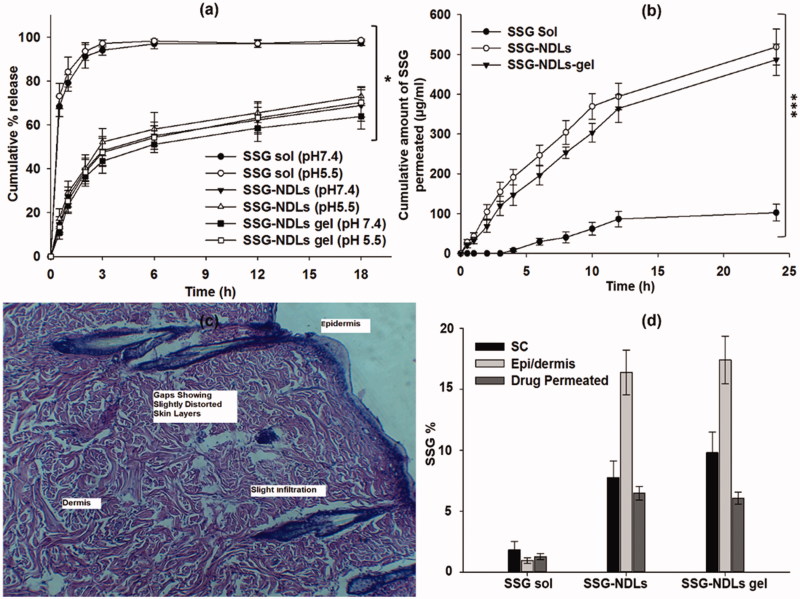
(a) *In vitro* drug release study in PBS (pH 7.4) and sodium acetate (pH 5.5) buffers. (b) *Ex vivo* permeation study in PBS of pH 7.4. (c) Histopathological study of the skin patch treated with plain SSG, showing gaps and slight distorted skin layers. (d) Comparison of SSG percentage permeated and SSG retention in the stratum corneum and epidermis/dermis layers of the skin treated with plain SSG, SSG-NDLs, and SSG-NDLs gel.

The release rate of a drug from lipid-based formulation depends on particular composition, vesicle size distribution, and relative proportion of the vesicle components (Cipolla et al., 2014). The drug release study suggests the duration of drug availability and release characteristics regulate the amount of drug that could possibly penetrates different layers of the skin. The drug release from the SSG-NDLs and SSG-NDLs gel formulation was significantly (*p* < .05) lower as compared to SSG solution. The drug release study displayed that 48% and 43% drug was released from SSG-NDLs and SSG-NDLs gel formulation, respectively, in the first 3 h while 94% of the drug was released from the drug solution in the same time period (Figure 1(a)). The outcomes of study revealed that the SSG-NDLs displayed burst release in the initial hours, possibly due to drug desorption and release from the vesicle surface, followed by slow release confirming a controlled release pattern at both pH 7.4 and pH 5.5 media. This displayed that developed NDLs had the capability to sustain the drug release over an extended period of time and avoid frequent application of drug. Moreover, neutral or acidic pH of the media had no effect on the drug release from the SSG-NDLs and SSG-NDLs gel, as reported previously (Want et al., 2017).

To understand the drug release model and mechanism responsible for SSG release from NDLs, different kinetic models were fitted, as shown in Supplemental Table S3. *R*^2^ values of these models showed highest linearity with Korsmeyer–Peppas model. Moreover, diffusion exponent (*n*) values of NDLs were lower than 0.4 which indicated that release of SSG from formulations was diffusion controlled.

### *Ex vivo* permeation and drug deposition studies

The permeation profile of all the formulations is shown in Figure 1(b). It was noted that a very small fraction (<1.28%) of plain drug had permeated through the skin from SSG solution over 24 h. The plain SSG finds it difficult to cross the SC because of its properties as explained previously. The crystalline nature of SC is the key barrier for permeation of hydrophilic drugs and needs to be revised using drug vehicles with latent ability. The permeation study failed to display any SSG content till 4 h from the SSG solution. However, beyond 4 h, an increase in skin permeation was seen which may be due to skin irritation profile of SSG (Oliveira et al., 2011). To verify this, the skin section used for permeation study was recovered and histopathological study was conducted (Figure 1(c)). As expected, gaps were seen in the SC region which may result in the permeation of plain drug. It was observed that 1.28%, 6.48%, and 6.08% of the total drug was permeated from the SSG solution, SSG-NDLs, and SSG-NDLs gel, respectively. Important parameters of permeation are mentioned in Supplemental Table S4. It was also noted that the cumulative % permeation of SSG constantly increased with the passage of time from all nano-formulations. The better skin permeation of NDLs can be described by the ability of NDLs to penetrate through the small openings (Cevc, 1996). The driving force required for the shape transformation is derived from trans-epidermal hydration gradient generated by difference in water content between skin surface and epidermis (Avadhani et al., 2017). Similar observation had been previously reported where NDLs increased the skin retention along with improvement in the penetration rate due to transcutaneous hydration gradient (Avadhani et al., 2017).

The NDLs also displayed high skin retention due to characteristic flexibility of the lipid bilayers which helps in squeezing across the skin layers and maintaining themselves trapped in the dermis (Dubey et al., 2007). Figure 1(d) compares the total percentage of SSG permeated along with drug deposited in the SC and epidermis/dermis region. The nano-formulations significantly increased the SSG deposition in the SC and epidermis/dermis compared to drug solution. The SSG deposition was more in epidermis/dermis region compared with the SC. NDLs promote passage of drug through the skin and control its permeation, supplying therapeutic level of SSG at the local site (dermis) for an extended period and reducing systemic effects (Dubey et al., 2007).

### *Ex vivo* penetration study with fluorescent dye

**Figure 2.**
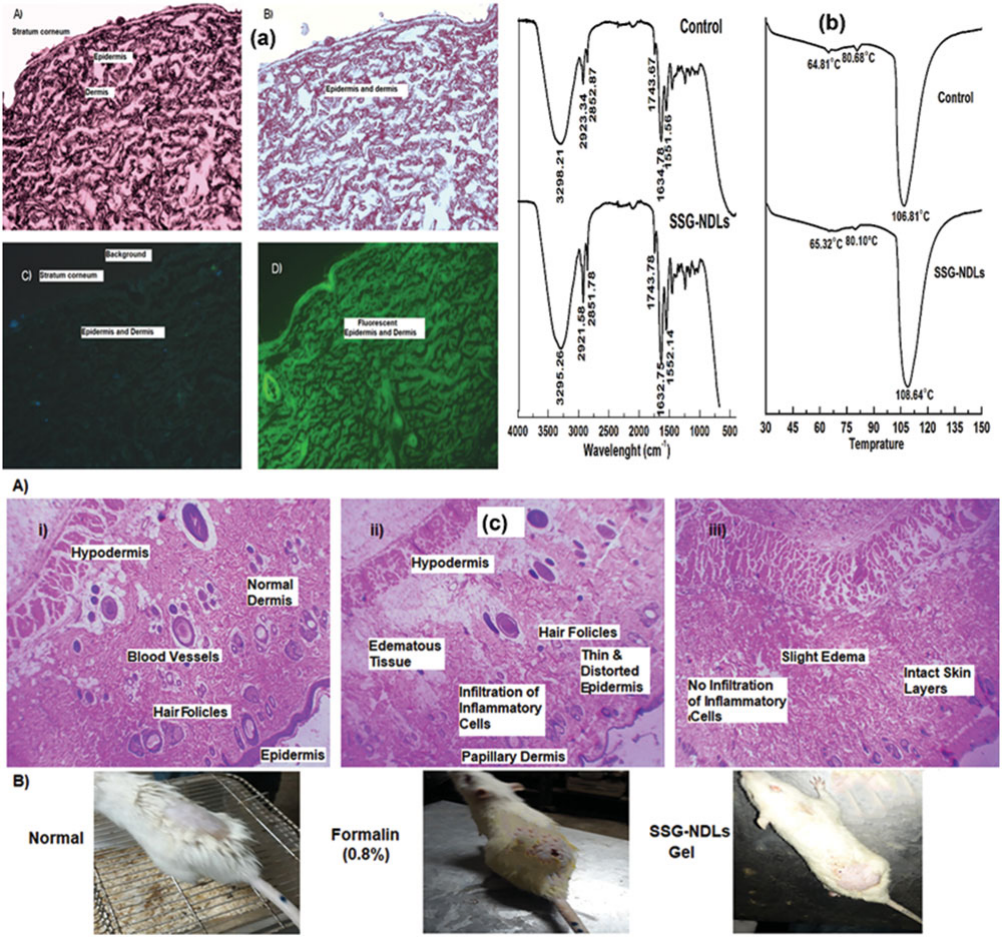
(a) Images observed under normal light microscope (A and B) and corresponding fluorescent microscope (C and D) of normal (A and C) skin and FITC/SSG-NDLs treated (B and D) skin sections after 24 h topical application. (b) FTIR spectra and DSC thermogram of untreated and SSG-NDLs gel treated epidermis. (c) Hematoxylin and eosin stained cross sectional images of different skin sections of (i) blank group, (ii) formalin (0.8%), and (iii) SSG-NDLs gel groups.

The skin penetration of FITC/SSG-NDLs was visualized under normal light and fluorescence microscope (Figure 2(a)). The incorporation of FITC resulted in a strong fluorescent marking of dermis in comparison to the control group. The fluorescence seemed to be fairly distributed and results indicated that NDLs was able to deliver a handsome amount of SSG deep in the skin, verifying the observations of drug deposition study.

### Evaluation of skin structure after SSG-NDLs treatment

Permeation enhancers are mostly used to increase percutaneous absorption which may decrease the barrier function of SC by fluidization and destabilization of the skin lipids. NDLs penetrate into intercellular region *via* shape deformation mechanism and display no significant effect on the barrier function of SC. In this study, the conservation of epidermal structure was verified by ATR-FTIR and DSC. SC may produce bands at various wavenumbers, most important peaks are the symmetric and asymmetric stretching vibrations at 2850 and 2920 cm^−1^, respectively, of the hydrocarbon chains of lipid. The bands around 1550 and 1650 cm^−1^ are due to vibrations of amide II (N–H and C–N stretching) and amide I (C = O stretching) bonds in SC protein, respectively (Obata et al., 2010), while a minor molecular vibration is observed around 1740 cm^−1^ due to stretching vibration of a small ester band. These vibrations in control epidermis was observed at 2852.87, 2923.34, 1551.56, 1634.78, and 1743.67 cm^−1^ while SSG-NDLs treated epidermis displayed these vibrations at 2851.78, 2921.58, 1552.14, 1632.75, and 1743.78 cm^−1^, respectively. It was observed that there was minute difference in molecular vibrations between the control and SSG-NDLs treated epidermis (Figure 2(b)).

The endothermic transitions of normal epidermis were observed at 64.81, 80.65, and 106.81 °C in connection with melting of intercellular lipids, protein associated lipids, and denatured proteins, respectively. When the intercellular lipid structure is disturbed, lipid transition temperature around 65 °C is reduced considerably (Dreher et al., 1997). The treated epidermis displayed three endothermic transitions at 65.32, 80.1, and 108.64 °C (Figure 2(b)), suggesting the lack of noticeable changes in the lipid organization. Taken together, the outcomes suggested that the penetration of NDLs was due to elastic and shape transformation potential without having substantial structural changes in SC.

### *In vivo* skin irritation and histopathological study

The mean PII score for plain SSG solution and SSG-NDLs gel was found to be 3.4 and 0.6, respectively. The PII score of SSG-NDLs gel was significantly lower (*p* < .05) than standard irritant (Supplemental Table S5). This showed that no significant erythema or edema was observed on the formulation treated skin. These results were further verified by the histopathological study which showed that the treated skin patch did not display any infiltration of inflammatory cells or had damaged epidermis and is similar to normal untreated skin in terms of skin morphology (Figure 2(c)). This may be due to high penetration of intact vesicles which may minimized the direct contact of SSG molecules with the skin. It can be inferred that developed NDLs-gel was less irritant, well tolerable and safe for delivery across skin.

### Stability study

Lipid-based system has a natural tendency to aggregate or fuse during storage, which direct changes in VS, PDI, ZP, and EE. In this study, the VS, PDI, ZP, and EE variations at 25 °C were prominent than changes observed at 4 °C, but overall SSG-NDLs did not show any major changes in the physicochemical parameters (Table 2). As explained previously, this may be due to higher zeta potential of dialyzed vesicles in comparison to un-dialyzed vesicles. The high value of zeta potential signified high stability with low aggregation tendency (Honary & Zahir, 2013). To verify the results, the un-dialyzed formulation was also subjected to stability study in terms of VS, PDI, and ZP parameters at 25 °C (Table 2). It was observed that un-dialyzed vesicles produced marked difference in the physicochemical parameters when stored at 25 °C. It can be inferred from these findings that dialysis might confer physical stability to the vesicles.

**Table 2.** Storage stability of dialyzed and un-dialyzed SSG-NDLs and SSG-NDLs gel for 60 days.

Day	Size (nm)		PDI		Zeta potential (mV)		Entrapment efficiency (%)		Precipitation		Phase separation		Un-dialyzed vesicles		
													Size (nm)	PDI	Zeta potential (mV)
	4 °C	25 °C	4 °C	25 °C	4 °C	25 °C	4 °C	25 °C	4 °C	25 °C	4 °C	25 °C	25 °C	25 °C	25 °C
0	195.1 ± 3.67	195.1 ± 3.67	0.158 ± 0.0145	0.158 ± 0.0145	−32.8 ± 0.88	−32.8 ± 0.88	35.26 ± 1.8	35.26 ± 1.8	No	No	No	No	187.9 ± 3.5	0.143 ± 0.0422	−7.66 ± 0.6
10	195.5 ± 3.19	198.4 ± 4.13	0.154 ± 0.0176	0.159 ± 0.0954	−32.3 ± 0.70	−31.8 ± 0.41	34.92 ± 1.4	34.68 ± 1.9	No	No	No	No	203.7 ± 1.6	0.161 ± 0.0648	−6.29 ± 0.5
15	197.4 ± 2.74	205 ± 3.27	0.164 ± 0.0096	0.166 ± 0.0415	−31.9 ± 0.82	−29.3 ± 1.04	34.16 ± 1.9	32.34 ± 1.1	No	No	No	No	212.2 ± 2.9	0.173 ± 0.0781	−4.91 ± 0.7
30	199.1 ± 1.52	213.2 ± 3.77	0.167 ± 0.0133	0.17 ± 0.0275	−31.0 ± 0.91	−29.0 ± 0.52	33.71 ± 0.9	31.22 ± 2.3	No	No	No	No	223.5 ± 1.1	0.211 ± 0.01628	−4.53 ± 0.4
45	201.7 ± 3.58	217.9 ± 2.81	0.173 ± 0.0232	0.193 ± 0.0153	−30.6 ± 0.50	−27.6 ± 0.79	31.28 ± 1.7	29.77 ± 1.5	No	No	No	No	237.5 ± 2.5	0.252 ± 0.0373	−3.27 ± 0.7
60	205.3 ± 2.82	223.5 ± 3.55	0.181 ± 0.0147	0.211 ± 0.0913	−29.9 ± 0.65	−26.7 ± 0.31	31.05 ± 2.1	28.93 ± 1.3	No	No	No	No	247 ± 3.8	0.276 ± 0.0843	−2.83 ± 0.6

### Macrophages cytotoxicity assay

**Figure 3.**
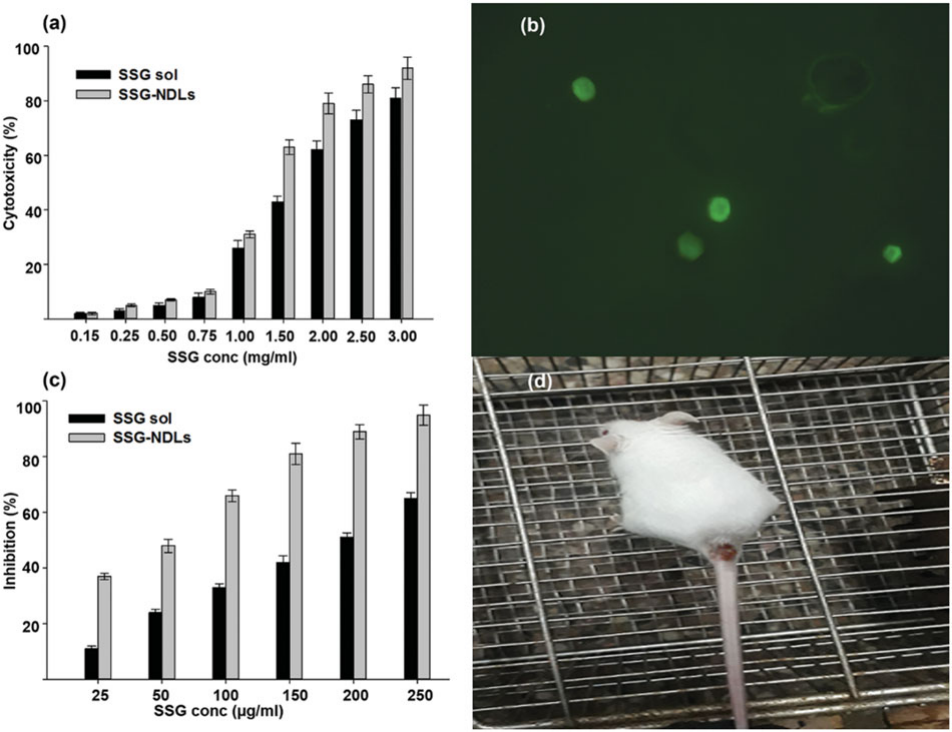
(a) Cytotoxicity potential of SSG solution and SSG-NDLs on the mice peritoneal macrophages. (b) Uptake of FITC/SSG-NDLs in mice peritoneal macrophages after 30 min incubation. (c) Inhibitory effect of different concentrations of SSG solution and SSG-NDLs on amastigote growth. (d) *In vivo* BALB/c infection model of cutaneous leishmaniasis.

*In vitro* cytotoxicity assay was performed to establish whether the drug concentration used on amastigotes was toxic to the macrophages itself or not. It was expected that nano-formulations would be more biocompatible because drug entrapment inside the vesicles would minimize the direct exposure of SSG to the normal cells. However, the assay revealed that the CC_50_ values for SSG and SSG-NDLs were 1.65 and 1.3 mg/ml, respectively. The comparison of CC_50_ values indicated that there was 21.2% reduction in CC_50_ of SSG-NDLs in comparison to free SSG as shown in Figure 3(a). Generally, the active form of antimony (Sb-III) is toxic to the body and therefore the antimonial preparations come in the form of Sb-V, complexed in the form of sodium stibogluconate and meglumine. Once inside the macrophages, Sb-V complexes are reduced to Sb-III and act against amastigotes which may produce cytotoxic effects (Borborema et al., 2011). This reduction in CC_50_ value verified our claim of higher cellular uptake of SSG-NDLs in comparison to plain SSG and might be the possible reason for this reduction.

### Qualitative and quantitative macrophage uptake study

The result of qualitative uptake study showed that fluorescent NDLs was endocytosed within 30 min of incubation and intense intracellular fluorescence was witnessed in the cytosol of PMs (Figure 3(b)). The simple SSG-NDLs, which was used as a control, generated no fluorescence when placed with macrophages as observed under a fluorescent microscope (Supplemental Figure S4). The qualitative estimation of macrophage internalization was then confirmed through quantitative macrophage uptake study. The results showed that the NDLs group displayed a handsome amount of SSG, that is, 81.69 ± 1.73 µg/2 × 10^4^ macrophages, while only 8.73 µg ± 0.89 µg/2 × 10^4^ cells was detected in the plain SSG group. The NDLs cell internalization was almost 10-fold higher than SSG solution. The effect was obvious that SSG solution undergoes distribution rather than specifically internalization by the macrophages.

It was expected that anti-leishmanial efficacy and potential of SSG could be significantly improved when entrapped within NDLs due to favorable vesicle characteristics and successful localization of vesicles in the infested macrophages, which constitute the preferred site of action for anti-leishmanial drugs (Borborema et al., 2011). Moreover, macrophages may also act as a secondary drug depot due to internalization of drug loaded particles which may enhance drug availability against parasites residing within them (Moosavian Kalat et al., 2014). A higher uptake of pentavalent antimony by *Leishmania* infected macrophages was observed using negatively charged liposomes. This resulted in higher *in vitro* and *in vivo* efficacies against *Leishmania* (Tempone et al., 2004).

### Anti-leishmanial activity on intramacrophage amastigote model

The entry of promastigotes inside the macrophages involves formation of parasitophorous vacuoles and transmute into the immotile amastigotes. There was considerable reduction in the number of intramacrophage amastigotes upon treatment (Figure 3(c)). The IC_50_ of plain SSG solution and NDLs-SSG was 184.66 and 50.86 µg/ml which was considerably higher than CC_50_ value. Several studies showed that vesicles enhanced the anti-leishmanial activity of SSG when compared with plain SSG solution against *Leishmania* (Alving et al., 1978; Baillie et al., 1986). The parasiticidal potential of these vesicles was ascribed to better interaction with parasite membrane which may result in direct killing and creation of a strong host protective environment, requiring a very low SSG dose for the effective treatment. Moreover, the small size and presence of negative charge may stimulate the targetability of the vesicles, possibly due to ligand binding properties of macrophage scavenger receptors (Tempone et al., 2004), which may deliver vesicles to the phagolysosomes. Negatively charged vesicles may target the parasitophorous vacuoles inside macrophages by a mechanism similar to annexins, the hydrophilic proteins that reversibly bind with negatively charged phospholipids and behave as bridging molecules in the vesicle fusion process (Tempone et al., 2004).

It is considered that a drug or formulation is nontoxic and have promising activity when SI value is ≥10 (Nwaka & Hudson, 2006). The SI value of SSG solution and SSG-NDLs was 8.94 and 25.56, respectively. The SI value of NDLs was almost threefold higher than SSG solution. The SI value of SSG-NDLs had increased despite increased cytotoxicity of NDLs.

### *In vivo* efficacy of formulation in BALB/c infection model of CL

Although intramacrophage amastigote drug assay had proven to be an appropriate model for the prediction of *in vivo* drug activity however, the lack of biological relevance (existence of many other types of cells and macrophage behavior under physiological or pathological conditions) might confound this supposition. Therefore, the efficacy of SSG-NDLs gel was evaluated in the BALB/c infection model of CL as shown in Figure 3(d). The average lesion size before treatment in the control and SSG-gel groups was 4.71 ± 0.39 and 4.89 ± 0.53 mm, respectively, which was further increased after treatment 5.18 ± 0.44 and 5.03 ± 0.36 mm, respectively. However, lesion size in the SSG-NDLs group was significantly reduced after treatment 1.92 ± 0.31 mm when compared with lesion size before treatment 4.83 ± 0.47 mm. Similarly, the infection intensity in the control and SSG-gel groups was marked as severe infection however, weak infection was noticed in the SSG-NDLs gel group. *In vivo* study exhibited that the therapeutic effect of SSG-NDLs formulation was significantly higher than simple SSG-gel because vesicles target intracellular parasites and inhibit rapid clearance of the drug (El Maghraby et al., 2008; Bavarsad et al., 2012).

## Conclusion

The stable SSG-NDLs based formulation with improved efficiency against *in vivo* infection model of CL in BALB/c mice was investigated for the targeted dermal delivery of SSG against CL. NDLs lack complications which are mostly seen in the intralesional/systemic SSG injection and create new opportunities for the topical delivery of anti-leishmanial drugs. The findings establish that SSG-NDLs was effective against amastigotes residing within macrophages and therefore proved to be an effective leishmanicidal formulation with minimum toxicity.

## Supplementary Material

supplementary_material.docx
