# Supplementary material for: Sodium stibogluconate loaded nano-deformable liposomes for topical treatment of leishmaniasis: macrophage as a target cell
Source: Drug Deliv. 2018 Aug 14;25(1):1595–606. doi: 10.1080/10717544.2018.1494222 (PMC6095017; doi:10.1080/10717544.2018.1494222)
Supplement: supplementary_material.docx [file IDRD_A_1494222_SM5252.docx]

**Sodium Stibogluconate loaded Nano-deformable Liposomes for Topical Treatment of Leishmaniasis: Macrophage as a target cell**

M. Junaid Dar, Fakhar-ud-Din, Gul Majid Khan*

# Supplemental Tables with captions

Table S1. Box Behnken design (BBD) for optimization of SSG-NDLs

| Factors | Levels | | | | |
| --- | --- | --- | --- | --- | --- |
|  | Low | Medium | | High |  |
|  | (-1) | (0) | | (+1) |  |
| Independent variables |  |  |  | | |
| X_1_: Sodium Stibogluconate concentration | 50 | 75 | 100 | | |
| (mg/ml) |  |  |  | | |
| X_2_: Amount of phospholipid | 200 | 300 | 400 | | |
| (mg) |  |  |  | | |
| X_3_: Percentage of edge activator | 5 | 10 | 15 | | |
| (% w/w) |  |  |  | | |
| Responses (dependent variables) | Constraints | | | | |
| Y_1_: Vesicle size (nm) |  |  | >100 | | |
| Y_2_: Entrapment Efficiency (%) |  |  | Maximize | | |

Table S2. Result of regression analysis for responses along with predicted and observed values for the optimized SSG-NDLs.

|  | Model | | Adequate precision | R^2^ | Adjusted R^2^ | Predicted R2 | p-value | Expected response | Observed response | Residual^a^ |
| --- | --- | --- | --- | --- | --- | --- | --- | --- | --- | --- |
| Y1: VS | | Quadratic | 13.969 | 0.9738 | 0.9267 | 0.7287 | 0.0019 | 197.196 | 195.1 | 2.096 |
| Y2: EE% | | Linear | 16.543 | 0.8780 | 0.8447 | 0.7599 | <0.0001 | 33.074 | 35.26 | -2.186 |

^a^ Residual = expected-observed

Table S3. Drug release kinetics of SSG-NDLs and SSG-NDLs gel in release media having different pH.

| Formulations | Kinetic models | | | | | | | |
| --- | --- | --- | --- | --- | --- | --- | --- | --- |
|  | Zero order (Q_t_ = Q_o_ +K_o_t) | | First order (ln Q_t_ = ln Q_o_ +K_1_ t) | | Higuchi (Q_t_ = K_H_ t_1/2_) | | Peppas (Q_t_/Q_∞_ = K_k_t_n_) | |
|  | K_o_ | R_2_ | K_1_ | R_2_ | K_H_ | R_2_ | R_2_ | n |
| SSG-NDLs (pH 7.4) | 4.90 | 0.086 | 0.13 | 0.613 | 19.29 | 0.832 | 0.958 | 0.312 |
| SSG-NDLs (pH 5.5) | 5.26 | 0.057 | 0.16 | 0.656 | 20.43 | 0.825 | 0.962 | 0.306 |
| SSG-NDLs gel (pH 7.4) | 4.62 | 0.193 | 0.10 | 0.616 | 17.81 | 0.858 | 0.949 | 0.334 |
| SSG-NDLs gel (pH 5.5) | 5.02 | 0.203 | 0.13 | 0.683 | 19.328 | 0.869 | 0.960 | 0.334 |

Q_t_ = Amount of drug released in time t, Q_o_ = Initial amount of drug, Q_∞_ = total amount of drug dissolved when the formulation is exhausted, N = Diffusion exponent, whereas K_o_, K_1_, K_H_ and K_k_ are rate constants for zero order, first order, Higuchi and Peppas respectively.

Table S4. Retention and permeability parameters obtained from *ex vivo* permeation and drug deposition studies.

| Formulations | Total drug permeated in 24 h Q (µg/cm^2^)^a^ | J_max_ (µg/cm^2^/h)^a^ | Enhancement ratio | Total drug deposited in 24 h (%)^a^ |
| --- | --- | --- | --- | --- |
| SSG Solution | 102.42 ±21.24 | 4.27 ±0.89 | 1 | 2.78 ±0.91 |
| SSG-NDLs | 518.61 ±44.84 | 21.61 ±1.87 | 5.06 | 24.15 ±3.20 |
| SSG-NDLs-gel | 486.63 ±39.53 | 20.28 ±1.65 | 4.75 | 27.22 ±3.62 |

^a^Each value represents average ±SD of three determination (n = 3).

Table S5. Comparative primary irritancy index after standard irritant and SSG-NDLs gel application on rats.

| Time | Blank | |  | Formalin (0.8%) | |  | SSG-NDLs gel | |
| --- | --- | --- | --- | --- | --- | --- | --- | --- |
|  | Edema | Erythema |  | Edema | Erythema |  | Edema | Erythema |
| 0 h | 0 | 0 |  | 0 | 0 |  | 0 | 0 |
| 1 h | 0 | 0 |  | 1 | 1 |  | 0 | 0 |
| 24 h | 0 | 0 |  | 3 | 3 |  | 1 | 1 |
| 48 h | 0 | 0 |  | 3 | 2 |  | 0 | 1 |
| 72 h | 0 | 0 |  | 2 | 2 |  | 0 | 0 |
| Mean ± SD | 0 ± 0 | 0 ± 0 |  | 1.8 ± 1.17 | 1.6 ± 1.02 |  | 0.2 ± 0.4 | 0.4 ± 0.4 |
| PII | 0 | |  | 3.4 | |  | 0.6 | |

Where PII is primary irritancy index

Erythema Formation: 0: No erythema, 1: Very slight erythema (barely perceptible), 2: Well defined erythema, 3: Moderate to severe erythema, 4: Severe erythema (beet redness)

Edema Formation: 0: No edema, 1: Very slight edema (barely perceptible), 2: Slight edema (edges of area defined by definite raising), 3: Moderate edema (Raised approximately 1 mm), 4: Severe edema (raised more than 1 mm and extending beyond the area of exposure).

# Supplemental Figures

Figure S1


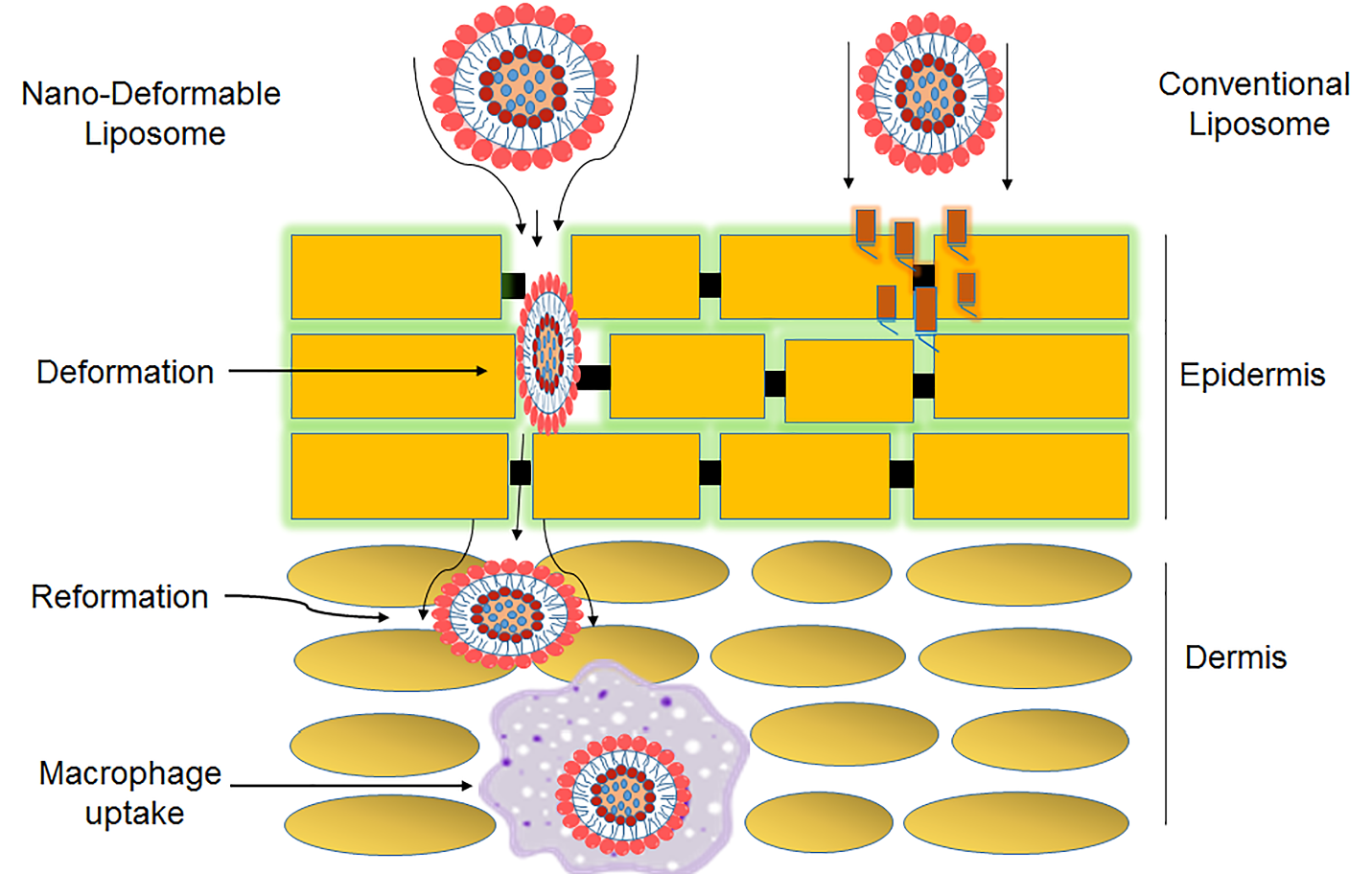


Figure S2


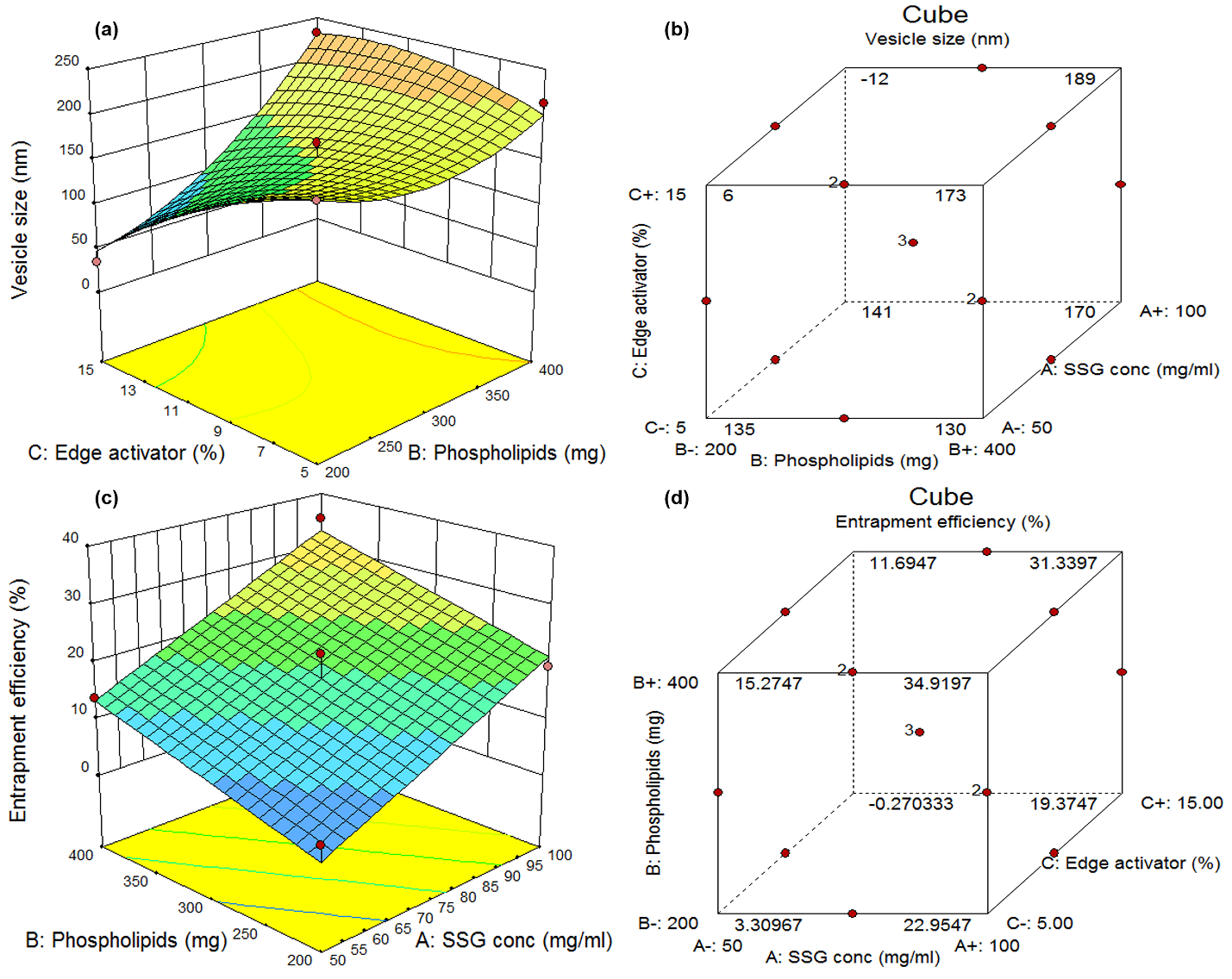


Figure S3


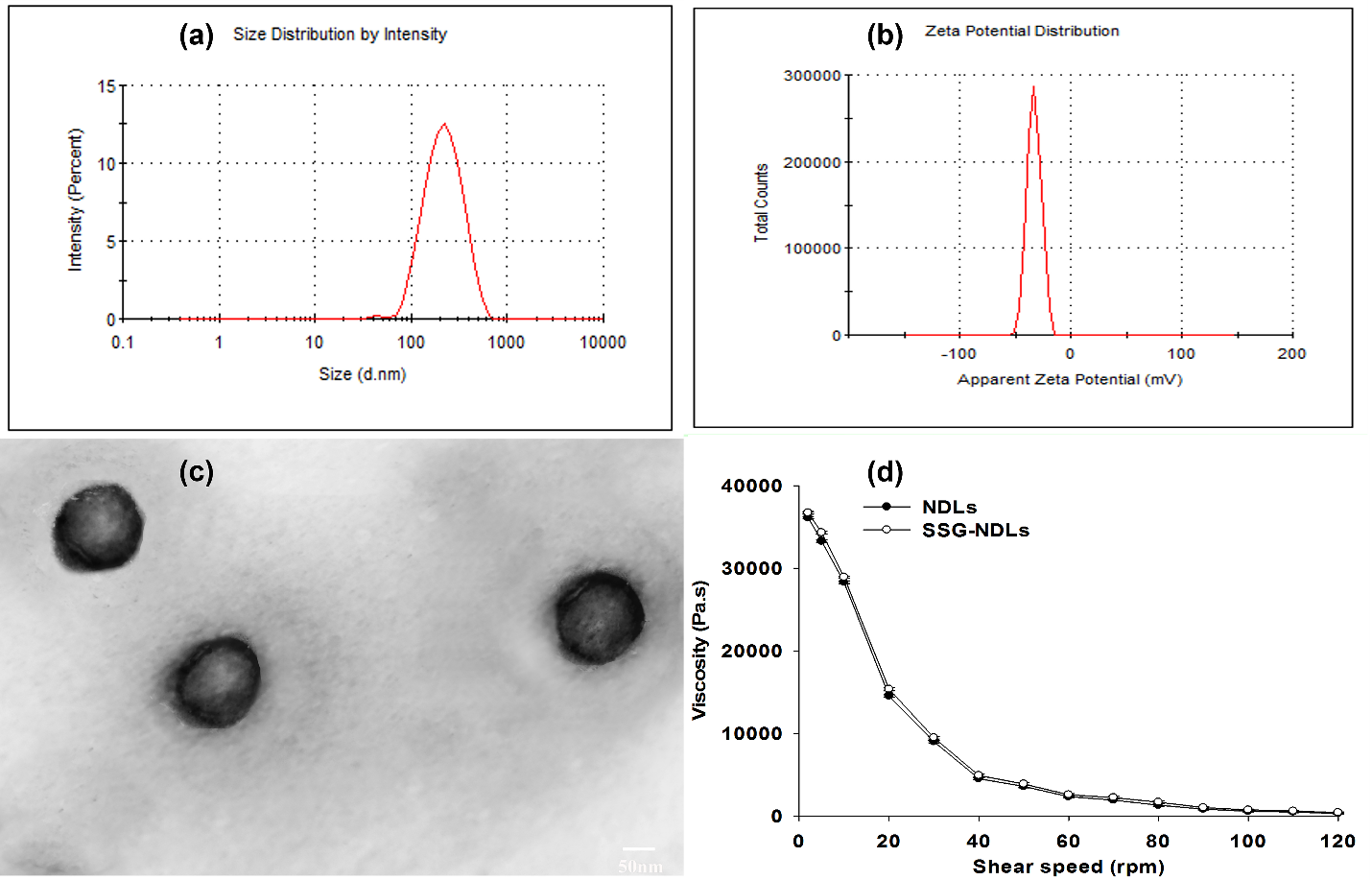


Figure S4


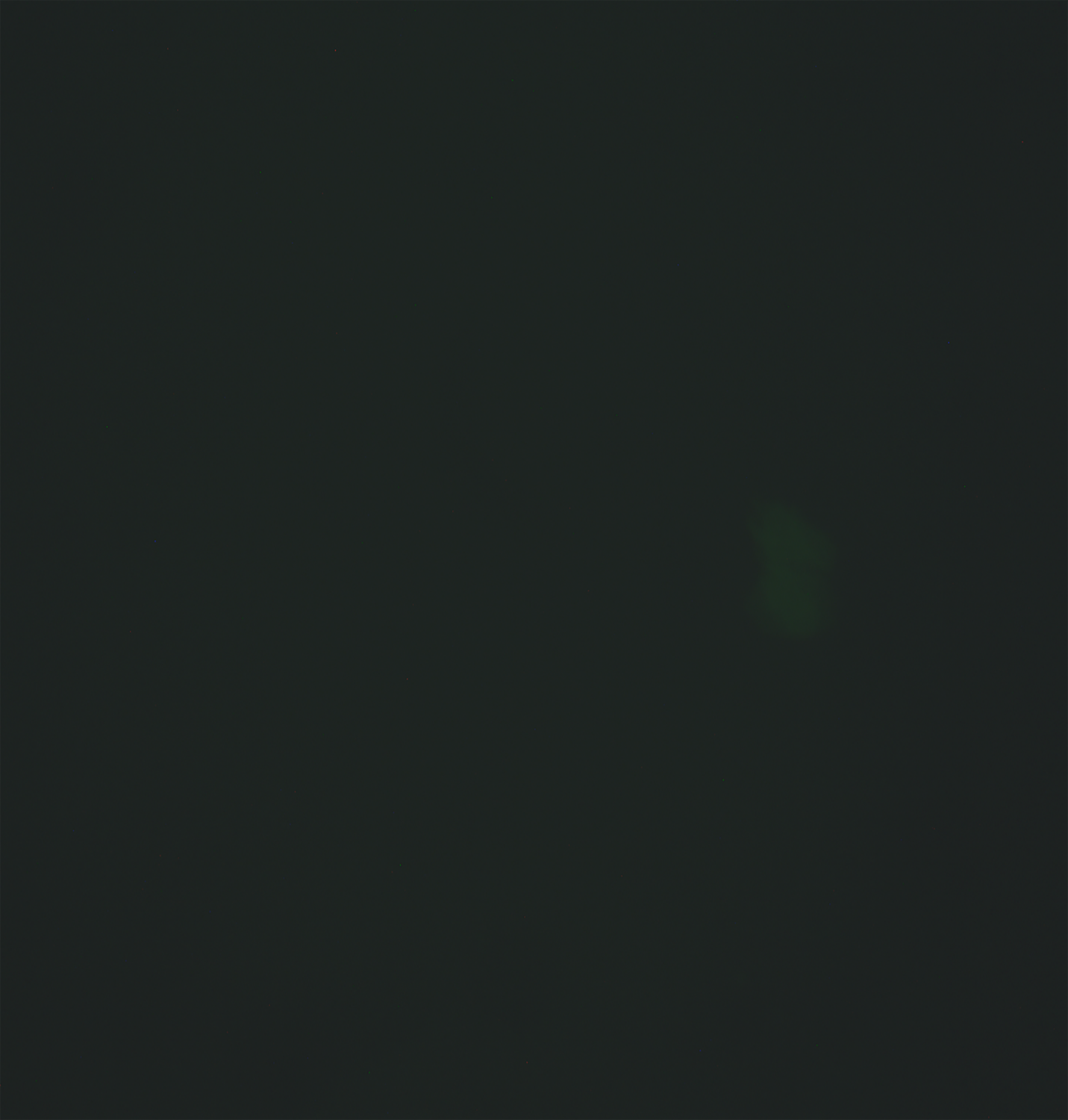


# Supplemental Figures captions

Figure S1. Diagrammatic illustration of mechanism governing the SSG-NDLs permeation in the skin via deformation/reformation mechanism and uptake by dermal macrophages.

Figure S2. Response surface 3D plots and cube plots for the effect of independent variables on (a) vesicle size and (b) entrapment efficiency.

Figure S3. (a) Vesicle size distribution, (b) zeta potential, (c) TEM image of optimized SSG-NDLs, and (d) flow curves of blank NDLs and SSG-NDLs gel achieved by plotting shear speed against respective viscosity.

Figure S4. The simple SSG-NDLs generated no fluorescence when placed with macrophages, as observed under a fluorescent microscope.
